# Supplementary material for: MacGyvered Multiproperty Materials Using Nanocarbon and Jam: A Spectroscopic, Electromagnetic, and Rheological Investigation
Source: J Funct Biomater. 2022 Jan 10;13(1):5. doi: 10.3390/jfb13010005 (PMC8788530; doi:10.3390/jfb13010005)
Supplement: Supplementary file 1 [file jfb-13-00005-s001.zip › jfb-1494653-supplementary.pdf]

Supplementary Materials

MacGyvered Multiproperty Materials Using Nanocarbon and Jam: A Spectroscopic, Electromagnetic and Rheological Investigation

Antonino Cataldo [1,2,3,*,†], Matteo La Pietra [3,†], Leonardo Zappelli [2], Davide Mencarelli [2], Luca Pierantoni [2] and Stefano Bellucci [3]

Scanning electron microscopy (SEM)

The most representative SEM micrographs of the surface of JAM, JAM-MWCNT and JAM-GNP nanocomposites are shown in Figures S1–S5.

The micrograph in Figure S1. shows the morphology of JAM hydrogel, which is characterized by an amorphous structure and a smooth and thickened surface.

The 0.05% CNT/jam composite is shown in the micrograph in Figure S2.

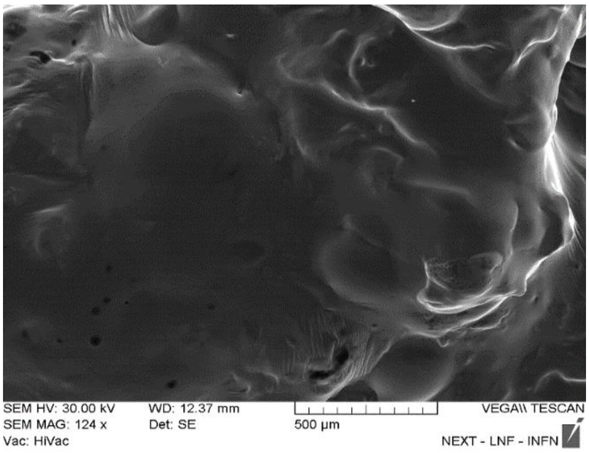

Figure S1. SEM micrograph of pure matrix.

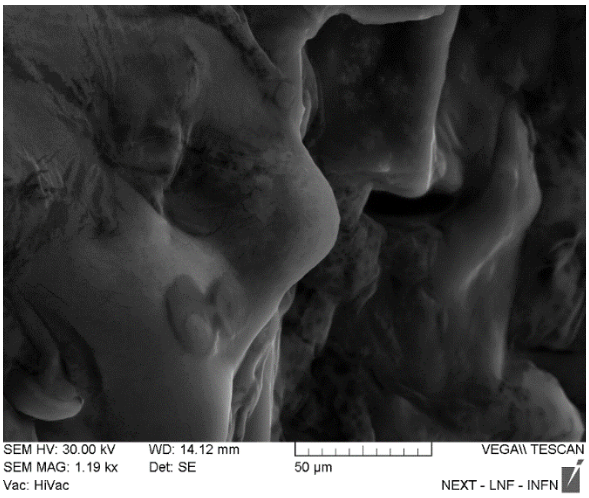

Figure S2. SEM micrograph of 0.05%$_{w/w}$ CNT /jam composite.

Adding a small quantity of filler to JAM matrix does not make substantial changes to the starting structure. However, the appearance of small lumps on the surface can be noted.
The 2.5% CNT/jam composite is shown in the micrograph in Figure S3.

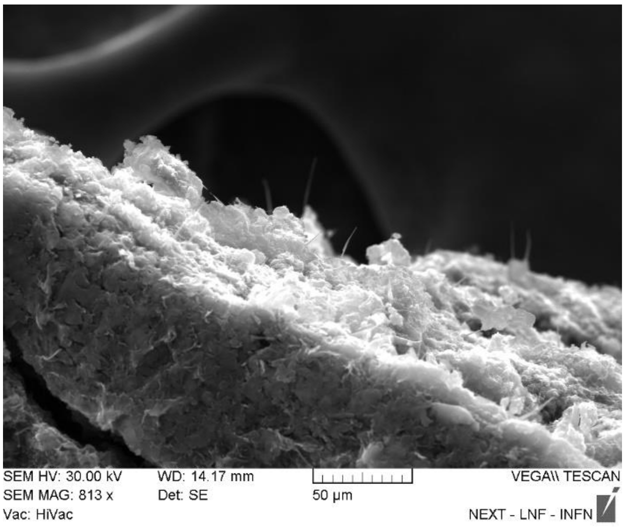

Figure S3. SEM micrograph of 2.5%$_{w/w}$ CNT/jam composite.

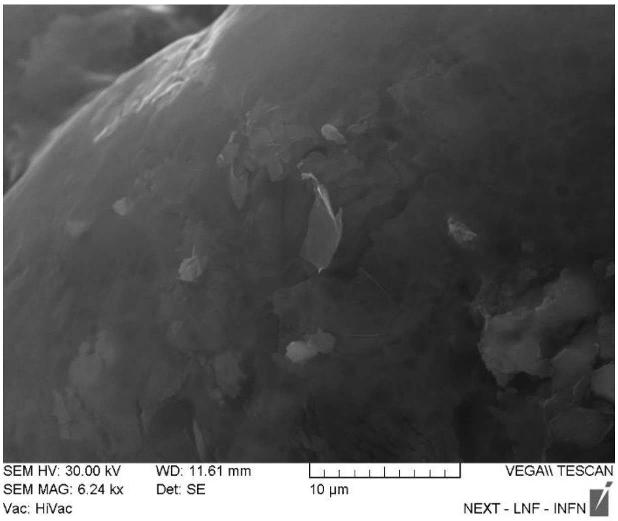

Figure S4. SEM micrograph of GNP/jam composite, 1.5%$_{w/w}$.

A greater quantity of filler into the JAM matrix makes a radical change of the starting structure: the previously smooth surface is characterized by a high roughness. This is probably due to the exceeded of percolation threshold, which led to a significant change of the starting structure.
The 0.05% GNP/jam nanocomposite is shown in the micrograph in Figure S5.

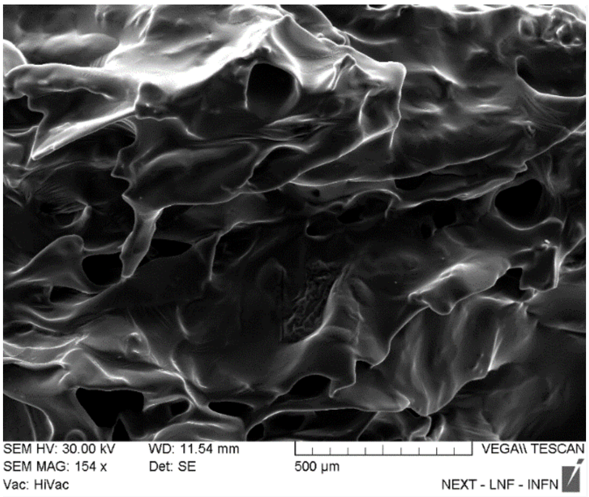

Figure S5. SEM micrograph of GNP/jam composite 0.05%w/w.

It is possible to observe a structure characterized by a jagged surface.
The 1.5% GNP/jam nanocomposite is shown in the micrograph in Figure S4.
The surface has a high roughness, this modification of the base structure is probably due to exceeded of the percolation threshold.
Furthermore, in addition to the changing in the surface morphology, SEM characterization reveals a low degree of agglomeration of the filler in the matrix.

Impendance measurements

The accuracy of MFIA 5 MHz Impedance Analyzer (Zurich Instruments) in the range observed is extremely high. As it is possible checked from the diagrams, the errors in the module of impedance is around 0.05-0.1%; the errors in the phase of impedance is at maximum 100mdeg.

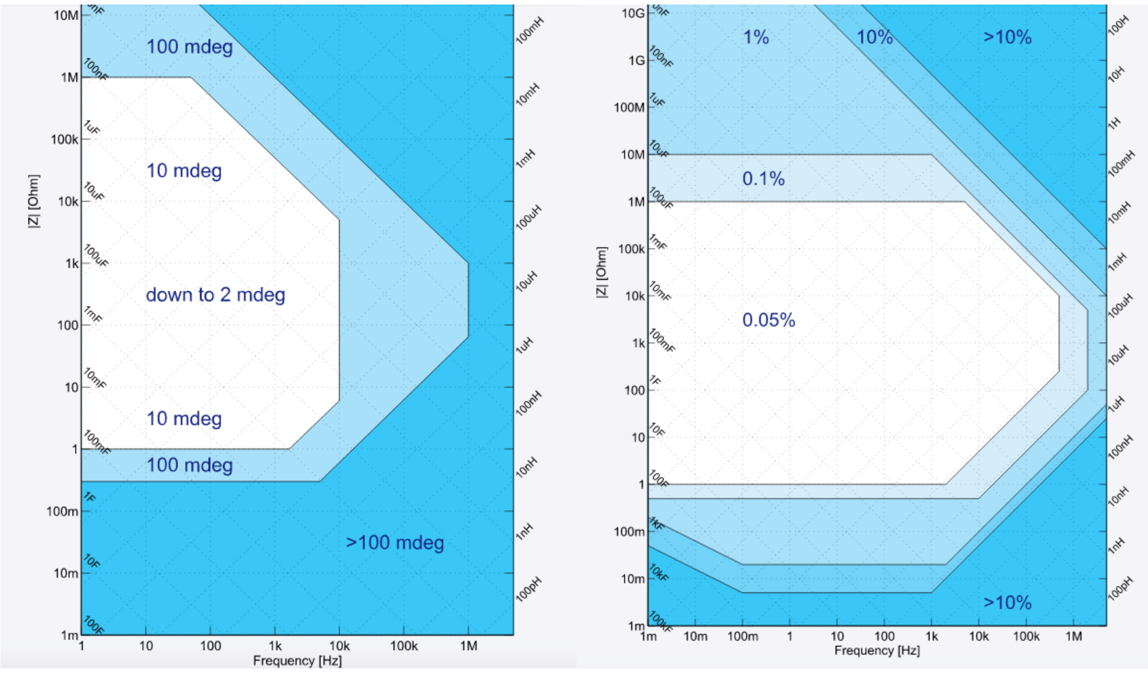

Figure S6. Errors chart of MFIA 5 MHz Impedance Analyzer.

Table S1. R2 values of fitting using different lumped circuits on CNT/jam nanocomposite specimens.

	**jam**		**CNT 0.05%**		**CNT 0.25%**		**CNT 0.5%**		**CNT 0.75%**		**CNT 1%**		**CNT 1.5%**	
	R2 ∣Z∣	R2 φ(Z)	R2 ∣Z∣	R2 φ(Z)	R2 ∣Z∣	R2 φ(Z)	R2 ∣Z∣	R2 φ(Z)	R2 ∣Z∣	R2 φ(Z)	R2 ∣Z∣	R2 φ(Z)	R2 ∣Z∣	R2 φ(Z)
**R(RC)**	0.997075	0.980132	0.989371	0.937861	0.989888	0.935752	0.985852	0.924949	0.979513	0.90973	0.983092	0.935087	0.730619	-0.19632
**$R(RC)_2$**	0.999841	0.990138	0.998709	0.982644	0.999237	0.9882	0.998902	0.986296	0.99749	0.976657	0.998169	0.982846	0.960127	0.632818
**$R(RC)_3$**	0.999986	0.997699	0.999875	0.997289	0.999908	0.997848	0.999866	0.997203	0.999634	0.994561	0.999703	0.994937	0.991622	0.88711
**$R(RC)_4$**	0.999998	0.999397	0.999978	0.999452	0.999976	0.999399	0.999972	0.999233	0.999912	0.998275	0.99993	0.998093	0.99806	0.968475

Table S2. R2 values of fitting using different lumped circuits on GNP/jam nanocomposite specimens.

	**jam**		**GNP 0.05%**		**GNP 0.25%**		**GNP 0.5%**		**GNP 0.75%**		**GNP 1%**		**GNP 1.5%**	
	R2 ∣Z∣	R2 φ(Z)	R2 ∣Z∣	R2 φ(Z)	R2 ∣Z∣	R2 φ(Z)	R2 ∣Z∣	R2 φ(Z)	R2 ∣Z∣	R2 φ(Z)	R2 ∣Z∣	R2 φ(Z)	R2 ∣Z∣	R2 φ(Z)
**R(RC)**	0.997075	0.980132	0.998046	0.956655	0.986395	0.921644	0.980356	0.828983	0.977398	0.69123	0.963798	0.348026	0.930038	0.256005
**$R(RC)_2$**	0.999841	0.990138	0.999903	0.992687	0.998057	0.971937	0.996041	0.898227	0.995083	0.797699	0.99185	0.585033	0.98204	0.457222
**$R(RC)_3$**	0.999986	0.997699	0.999983	0.998632	0.999421	0.984857	0.999082	0.959127	0.998899	0.929733	0.998146	0.831893	0.99686	0.807729
**$R(RC)_4$**	0.999998	0.999397	0.999988	0.999529	0.999861	0.996541	0.999835	0.988633	0.99978	0.977066	0.999479	0.94829	0.999406	0.948438

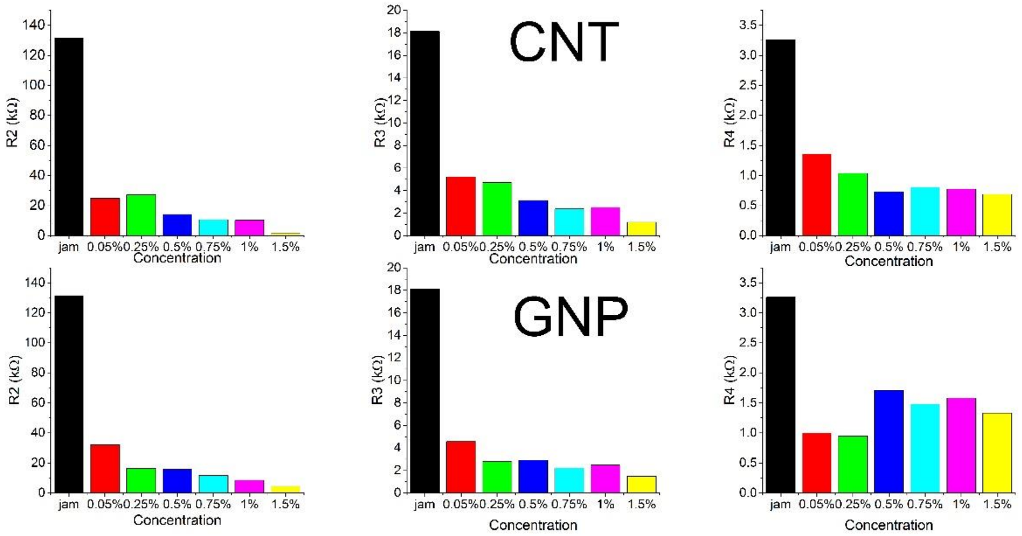

Figure S7. Best fitting results of resistences for CNT and GNP jam nanocomposite, (concentration 0% black, 0.05% red, 0.25% green, 0.5% blue, 0.75% cyano, 1% magenta, 1.5% yellow).

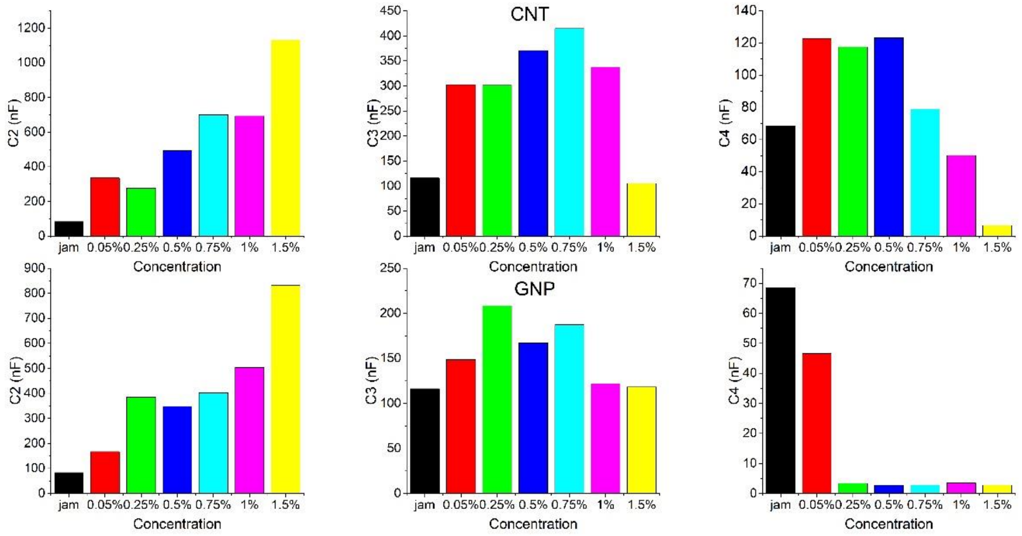

Figure S8. Best fitting results of capacitances for CNT and GNP jam nanocomposite, (concentration 0% black, 0.05% red, 0.25% green, 0.5% blue, 0.75% cyano, 1% magenta, 1.5% yellow).

Rheological measurements

Table S3. Yield zone for CNT/jam nanocomposite.

	LVE region limit (1/s)	*Flow point (1/s)*
Jam	0.046	0.336
CNT 0.05	0.039	0.317
CNT 0.5	0.016	0.247
CNT 0.75	0.018	0.144
CNT 1	0.016	0.212
CNT 1.5	0.011	0.356
CNT 2.5	0.0082	0.476

Table S4. Yield zone for GNP/jam nanocomposite.

	LVE region limit (1/s)	*Flow point (1/s)*
Jam	0.046	0.336
GNP 0.05	0.0034	0.178
GNP 0.5	0.0033	0.275
GNP 0.8	0.0024	0.149
GNP 1	0.0010	0.174
GNP 1.5	0.0017	0.092
